# Role of Vitamin A in Modulating Graft-versus-Host Disease

**Published:** 2018-06-03

**Authors:** Jianwei Zheng, Brian Taylor, Xiao Chen

**Affiliations:** 1 From the Division of Hematology & Oncology, Medical College of Wisconsin, Milwaukee, WI 53226, USA; 2 Department of Medicine, Medical College of Wisconsin, Milwaukee, WI 53226, USA; 3 Department of Oncology, Union Hospital of Fujian Medical University, Fuzhou, Fujian, China

**Keywords:** Vitamin A, Retinoic acid, Graft-versus-host disease, Allogeneic hematopoietic stem cell transplantation

## Abstract

Vitamin A is an essential micronutrient that participates in a wide range of biological processes. Retinoic acid (RA) is an active metabolite of vitamin A that functions as an immune regulator. Graft-versus-host disease (GVHD) is a major complication of allogeneic hematopoietic stem cell transplantation (HSCT). It is characterized by extensive inflammation arising from an alloimmune response involving various host and donor immune cells. Since vitamin A affects different immune cell lineages and regulates an array of immune responses, vitamin A, and more specifically retinoic acid, is likely to influence the incidence and/or severity of GVHD. Indeed, recent preclinical and clinical data support this concept. In this review, we briefly summarize recent advances in our understanding of the potential role of vitamin A in modulating GVHD risk after allogeneic HSCT.

## INTRODUCTION

### Vitamin A and its Metabolites

Vitamin A is a fat-soluble vitamin that plays an essential role in a wide range of biological processes. Retinoic acid (RA), the major metabolite of vitamin A, is vital for embryonic development, cell differentiation, proliferation, and apoptosis. RA is also important for visual function and immune homeostasis. Vitamin A is obtained from a diet containing carotenoids (vitamin A precursors found in plants) or retinyl esters (preformed vitamin A found in animal products) [1]. Retinyl esters are the primary storage form of vitamin A in the body while carotenoids are absorbed in the intestines and can be converted to retinol. RA is synthesized from vitamin A in two biological steps. The first step is the conversion of retinol into retinal by ubiquitously expressed alcohol dehydrogenase enzymes. In the second step, retinal is irreversibly oxidized to RA by one of the three aldehyde dehydrogenase isoforms (RALDHs) known as RALDH 1, 2, and 3. The expression of RALDHs, in particular RALDH2, is restricted to certain cell populations in mucosal tissues with the unique capacity to produce RA [2]. In addition, it has been shown that three enzymes of cytochrome P450 family 26 (CYP26A1, CYP26B1, and CYP26C1) are mainly responsible for the breakdown of RA [3,4].

### Vitamin A and the Immune System

Vitamin A has long been recognized as an important factor for maintaining immune homeostasis. It has been well documented that vitamin A deficiency impairs anti-pathogen immunity and is associated with increased susceptibility to infections [5]. Most immunological effects of vitamin A are exerted by the active metabolite retinoic acid. RA binds to three isoforms (RARα, RARβ, and RARγ) of the retinoic acid receptor (RAR). Upon encounter with RA, RARs heterodimerize with retinoid X receptors (RXRs). These heterodimers bind to retinoic acid responsive elements (RARE) located in the promoter region of target genes and function to regulate gene transcription. The RXR family also has three members, namely RXRα, -β, and -γ. *9-cis*-RA binds RXRs preferentially, whereas both *all-trans*-RA and *9-cis*-RA are ligands of RARs [6,7]. The effects of RA signaling on the immune response is complicated and context dependent, sometimes resulting in contrasting results. The immunological outcomes are often determined by local RA levels, the RA receptors involved, the target cell type, and the presence or absence of other cytokine signals.

RA can act on many innate and adaptive immune cell lineages to regulate various immune responses [8–10]. One of the most important functions of RA is to control the migration of T cells to the intestines. Under steady state conditions, it has been demonstrated that RA enhances the expression of gut-homing molecules CCR9 and α4β7 on T cells, thus augmenting their gut tropism. In addition to facilitating homing of T cells to the gut, RA is also required to induce gut-homing IgA-secreting B cells [11]. Furthermore, RA plays a central role in CD4^+^ T cell polarization. RA inhibits the generation of Th17 cells in vitro. In the presence of TGF-β, RA facilitates the conversion of CD4^+^Foxp3^-^ T cells into CD4^+^Foxp3^+^ induced regulatory T cells (iTregs) [12]. RA also enhances the gut-homing capacity of regulatory T cells by up-regulating their expression of CCR9 and α4β7 [13]. In addition, RA can stabilize the function of natural regulatory T cells [14]. Therefore, RA is generally regarded as a molecule with immunoregulatory function under steady state conditions, given its ability to induce iTregs, stabilize nTregs, and suppress Th17 cells. These properties of RA are critical for maintaining intestinal homeostasis and inducing oral tolerance. It has been shown that vitamin A deficiency results in impaired oral tolerance, demonstrating the critical role of RA in this process [15,16]. On the other hand, emerging evidence suggests that RA can also have an immunostimulatory function under certain conditions. Hall and colleagues showed that RA is required for CD4 T cell activation and proinflammatory cytokine secretion in response to infection [17]. Pino-Lagos and colleagues used vaccination and skin allograft models to demonstrate that RA signaling controls CD4 T cell differentiation, effector function, and migration [18]. Both studies revealed an essential role of the RA-RAR-α axis in mediating effective T cell responses under inflammatory conditions. In addition, RA can even fuel inflammatory immune responses under certain pathogenic conditions. For example, in a murine model of celiac disease, RA was shown to act on dendritic cells (DCs) to promote Th1 polarization and inhibit Treg induction in the presence of the proinflammatory cytokine IL-15 [19]. Thus, RA can have pro-inflammatory or anti-inflammatory functions depending on the immune context.

RA is also well known for its effects on myeloid cell differentiation, survival, and function. *All-trans* RA is used clinically for treating patients with acute promyelocytic leukemia due to its ability to induce the terminal differentiation of leukemic cells. Retinoids have been reported to regulate the survival and maturation of human DCs, the most potent antigen presenting cells of the immune system [20]. Recent studies have also demonstrated an important role of RA in controlling the differentiation of DCs. Lack of RA or blockade of RA signaling changes the composition of DC subsets in the spleen and intestines in mice [21,22]. RA can also strongly influence the function of DCs. RA facilitates the induction of mucosal-type DCs both *in vitro* and *in vivo* [23,24]. Furthermore, RA can modulate the immunostimulatory function of DCs by regulating the expression of MHC and costimulatory molecules [25,26].

### Potential Role of Vitamin A in GVHD

Allogeneic hematopoietic stem cell transplantation (HSCT) is an effective treatment for many hematological malignancies and nonmalignant disorders. Each year, many patients diagnosed with leukemia, lymphoma, and multiple myeloma will receive an allogeneic stem cell transplant in an effort to cure their hematologic malignancy. Although the conditioning regimen consisting of radiation and chemotherapy may directly kill tumor cells, the curative potential of allogeneic HSCT relies on a donor T cell mediated graft-versus-leukemia effect [27]. Unfortunately, donor T cells can also target host tissues causing GVHD. GVHD is the major complication of allogeneic HSCT and limits the wider application of this treatment [28–30]. GVHD occurs when immunocompetent donor T cells recognize the genetically disparate host as foreign. Donor T cells become activated when they encounter these “foreign” host antigens, expand, and acquire effector functions against host tissues. The development of GVHD is a complicated process involving different types of immune cells. Largely based on animal studies, the pathophysiology of acute GVHD can be summarized in three sequential phases. In the first phase, cytokine release due to conditioning regimen activates host antigen presenting cells (APCs). Gut damage induced by radiation increases intestinal permeability and allows for the systemic translocation of bacterial products such as LPS that can amplify inflammation [31,32]. In the second phase, naïve donor T cells recognize alloantigen presented by host APCs and are activated within secondary lymphoid tissue [33,34]. Donor T cells differentiate and proliferate rapidly during this stage. They also up-regulate tissue homing molecules on their cell surface under the influence of activated APCs. Donor T cells expressing specific tissue homing molecules can then traffic to skin, liver, and the gastrointestinal tract, which are the main target tissues of GVHD. In the third phase, proinflammatory cytokines such as TNF-α, interferon-γ, and interleukin-1β secreted by donor CD4^+^ T cells as well as CD8^+^ cytotoxic T cells cause target tissue damage characteristic of GVHD. Among these three phases, T cell activation, differentiation, and migration take the central stage of GVHD pathogenesis. Therefore, any factors that can influence these T cell properties have the potential to affect GVHD risk after allogeneic HSCT.

Emerging evidence suggests that vitamin A can affect GVHD development and severity. Indeed, recent pre-clinical and clinical data support the concept that vitamin A and/or retinoic acid can modulate GVHD risk after allogeneic HSCT.

### Pre-clinical Studies

The GI tract is one of major target organs of acute GVHD [31]. To cause gut damage, donor T cells must first migrate to this tissue. Since one of the most important immunological functions of RA is to induce the gut tropism of T cells, RA is likely to affect donor immune cell trafficking after allogeneic HSCT. In a recent study, Koenecke et al. [35] reported the shift of GVHD target organ tropism of donor T cells by dietary vitamin A. Specifically, they found a significant reduction in the expression of gut-homing molecules α4β7 and CCR9 on donor T cells isolated from vitamin A-deficient (VAD) HSCT recipients compared to that of vitamin A-sufficient control mice. Consequently, the migration of donor T cells to the intestines was decreased and overall survival was improved in VAD mice. However, these mice eventually developed more severe hepatic damage and died from GVHD. They also found a significantly increased ratio of IFN-γ^+^CD4^+^/Foxp3^+^CD4^+^in VAD recipients compared to control mice. These studies demonstrated that dietary vitamin A levels can modulate GVHD mortality by influencing donor T cell migration to GVHD target organs after experimental allogeneic HSCT.

Subsequently, two independent groups reported that genetic inhibition of RAR-α signaling in donor T cells significantly reduced their alloreactivity and ability to cause lethal GVHD [36,37]. They made similar observations that the expression of gut-homing molecules CCR9 and α4β7 on donor T cells was decreased when RAR-α signaling was abrogated. This resulted in a decrease in number of donor effector T cells in the intestines of recipient mice. In contrast, enhancing RAR signaling by exogenous RA administration significantly increased GVHD-associated mortality in experimental HSCT models [36–38]. Our studies have also shown that RA administration increased the number of Tregs in the colon tissue [36]. However, the magnitude of Treg expansion was substantially less than that of pro-inflammatory effector T-cell populations and was insufficient to prevent pathological damage within the colon. Importantly, Aoyama and colleagues further found that vitamin A metabolism is upregulated in the intestine and mesenteric lymph nodes during GVHD [37]. In addition, genetic inhibition of RAR signaling skewed donor T cell differentiation toward a Th2 phenotype and favored the induction of Tregs [37]. These studies provide strong evidence that RAR signaling in donor T cells can control their polarization and migration after HSCT. Inhibiting this pathway could be an attractive approach to mitigate GVHD without compromising the graft-versus-leukemia effect.

Thus, we further examined the effects of donor vitamin A deficiency and pharmacological inhibition of donor T cell retinoic acid pathway on GVHD severity [39]. We found that chronic vitamin A deficiency modifies the composition of T cell subsets in donor mice and significantly reduces the capacity of their T cells to cause lethal GVHD. Importantly, administration of a pan-RAR antagonist to donor mice caused a transient inhibition of donor T cell RAR signaling resulting in reduced T cell alloreactivity and a reduction in their ability to cause GVHD [39]. These studies suggested that donor vitamin A deficiency may be a previously unrecognized non-genetic factor that can reduce GVHD risk. In addition, pharmacologic interference of RA/RAR signaling in donor T cells has the potential to mitigate GVHD after allogeneic HSCT. Furthermore, given the demonstrated effects of vitamin A/RA on myeloid cells, we are also actively investigating how RA pathway influences host and donor myeloid cell populations during GVHD.

### Clinical Studies

Emerging clinical studies have also supported the concept that vitamin A is a potential factor that modulates GVHD risk, although some of the results obtained so far are inconsistent with animal studies.

In a recent study [40], Lounder and colleagues reported that the incidence of intestinal GVHD was significantly increased in pediatric patients when vitamin A levels were below the median at day 30 after allogeneic HSCT. Specifically, they measured plasma vitamin A levels in more than 100 consecutive patients undergoing allogeneic HSCT. The median vitamin A level was found to be 1.3 ng/ml. Importantly, the risk of developing grades 2–4 GVHD was significantly increased in patients with a plasma vitamin A level below the median compared to those with levels above the median. The risk of intestinal GVHD was also significantly increased in these patients, which was associated with increased intestinal permeability but not plasma IL-22 levels. Furthermore, they found that vitamin A levels did not appear to affect I-FABP levels, indicating that vitamin A does not protect against mucosal injury associated with allogeneic HSCT. Interestingly, flow cytometry data showed increased expression of the gut-homing molecule CCR9 on CD8^+^ effector T memory cells in patients with vitamin A levels below the median.

In another interesting clinical study, Tong and colleagues investigated the association between serum vitamin A levels and the development of ocular GVHD [41]. They measured patient serum vitamin A levels before HSCT, 3 months after allogeneic HSCT, and during chronic GVHD. They found a strong correlation between low serum vitamin A levels and increased grade of ocular GVHD. Despite a relatively small sample size, this study suggested that serum vitamin A levels may affect the severity of ocular GVHD after allogeneic HSCT. However, it is still unclear whether vitamin A metabolism is altered in other GVHD target organs and whether this influences organ-specific GVHD.

Furthermore, Tong et al. found that treatment with vitamin A ointment improved ocular GVHD in vitamin A deficient patients, providing further evidence that vitamin A metabolism may be involved in the pathogenesis of ocular GVHD. It will be interesting to see if vitamin A supplementation can reduce intestinal GVHD in pediatric patients after allogeneic BMT [42]. Continued study involving adult HSCT patients is needed to provide more information about the potential role of vitamin A in regulating GVHD risk after allogeneic HSCT. Finally, since *all-trans* RA is not the only active metabolite of vitamin A, whether vitamin A can act through an RA-independent manner to modulate GVHD warrants further study.

## CONCLUSION

It is interesting that a common micronutrient such as vitamin A can modulate the severity of a systemic disease such as GVHD. Preclinical studies demonstrated that exogenous RA intensifies GVHD and vitamin A-deficient recipients show reduced intestinal GVHD after allogeneic HSCT [35–38]. Clinical studies, however, have indicated that lower levels of vitamin A have a detrimental effect on intestinal and ocular GVHD [40,41]. The reason for the discrepancy between mouse and human studies is currently unclear. However, such observations further demonstrate the complex and context-dependent effects of vitamin A/RA on alloimmune responses. More basic and clinical research is needed to define the precise mechanisms underlying vitamin A effects on alloimmunity and to explore clinically applicable approaches to prevent and/or treat GVHD by modulating vitamin A or the retinoic acid pathway.

## References

[1] Harrison EH (2005) Mechanisms of digestion and absorption of dietary vitamin A. Annu Rev Nutr 25: 87–103. 16011460 10.1146/annurev.nutr.25.050304.092614

[2] Kumar S, Sandell LL, Trainor PA, Koentgen F, Duester G (2012) Alcohol and aldehyde dehydrogenases: retinoid metabolic effects in mouse knockout models. Biochim. Biophys. Acta 1821: 198–205. 21515404 10.1016/j.bbalip.2011.04.004 PMC3161159

[3] Thatcher JE, Isoherranen N (2009) The role of CYP26 enzymes in retinoic acid clearance. Expert Opin Drug Metab Toxicol 5: 875–886. 19519282 10.1517/17425250903032681 PMC2730205

[4] Ross AC, Zolfaghari R (2011) Cytochrome P450s in the regulation of cellular retinoic acid metabolism. Annu Rev Nutr 31: 65–87. 21529158 10.1146/annurev-nutr-072610-145127 PMC3789243

[5] Stephensen CB (2001) Vitamin A, infection, and immune function. Annu Rev Nutr 21: 167–192. 11375434 10.1146/annurev.nutr.21.1.167

[6] Mark M, Ghyselinck NB, Chambon P (2006) Function of retinoid nuclear receptors: lessons from genetic and pharmacological dissections of the retinoic acid signaling pathway during mouse embryogenesis. Annu Rev Pharmacol Toxicol 46: 451–480. 16402912 10.1146/annurev.pharmtox.46.120604.141156

[7] Balmer JE, Blomhoff R (2002) Gene expression regulation by retinoic acid. J Lipid Res 43: 1773–1808. 12401878 10.1194/jlr.r100015-jlr200

[8] Hall JA, Grainger JR, Spencer SP, Belkaid Y (2011) The role of retinoic acid in tolerance and immunity.Immunity 35: 13–22. 21777796 10.1016/j.immuni.2011.07.002 PMC3418663

[9] Cassani B, Villablanca EJ, De Calisto J, Wang S, Mora JR (2012) Vitamin A and immune regulation: role of retinoic acid in gut-associated dendritic cell education, immune protection and tolerance. Mol Aspects Med 33: 63–76. 22120429 10.1016/j.mam.2011.11.001 PMC3246074

[10] Raverdeau M, Mills KH (2014) Modulation of T cell and innate immune responses by retinoic acid. J Immunol 192: 2953–2958. 24659788 10.4049/jimmunol.1303245

[11] Mora JR, Iwata M, Eksteen B (2006) Generation of gut-homing IgA-secreting B cells by intestinal dendritic cells. Science 314: 1157–1160. 17110582 10.1126/science.1132742

[12] Mucida D, Park Y, Kim G, Turovskaya O, Scott I, Kronenberg M, Cheroutre H (2007) Reciprocal TH17 and regulatory T cell differentiation mediated by retinoic acid. Sci 317: 256–260. 10.1126/science.1145697 17569825

[13] Benson MJ, Pino-Lagos K, Rosemblatt M, Noelle RJ (2007) All-trans retinoic acid mediates enhanced Treg cell growth, differentiation, and gut homing in the face of high levels of co-stimulation. J Exp Med 204: 1765–1774. 17620363 10.1084/jem.20070719 PMC2118687

[14] Zhou XH, Kong N, Wang J (2015) Cutting Edge: All-trans retinoic acid sustains the stability and function of natural regulatory t cells in an inflammatory milieu. J Immunol 185: 2675–2679. 10.4049/jimmunol.1000598 PMC3098624 20679534

[15] Nakamoto A, Shuto E, Tsutsumi R (2015) Vitamin A deficiency impairs induction of oral tolerance in mice. J Nutr Sci Vitaminol (Tokyo) 61: 147–153. 26052145 10.3177/jnsv.61.147

[16] Turfkruyer M, Rekima A, Macchiaverni P (2016) Oral tolerance is inefficient in neonatal mice due to a physiological vitamin A deficiency. Mucosal Immunol 9: 479–491. 26530133 10.1038/mi.2015.114

[17] Hall JA, Cannons JL, Grainger JR, Dos Santos LM, Hand TW, (2011) Essential role for retinoic acid in the promotion of CD4(+) T cell effector responses via retinoic acid receptor alpha. Immunity 34: 435–447. 21419664 10.1016/j.immuni.2011.03.003 PMC3415227

[18] Pino-Lagos K, Guo Y, Brown C, Alexander MP, Elgueta R, (2011) A retinoic acid-dependent checkpoint in the development of CD4**+** T cell-mediated immunity. J Exp Med 208: 1767–1775. 21859847 10.1084/jem.20102358 PMC3171100

[19] DePaolo RW, Abadie V, Tang F (2011) Coadjuvant effects of retinoic acid and IL-15 induce inflammatory immunity to dietary antigens. Nature 471: 220–224. 21307853 10.1038/nature09849 PMC3076739

[20] Geissmann F, Revy P, Brousse N, Lepelletier Y, Folli C, (2003) Retinoids regulate survival and antigen presentation by immature dendritic cells. J Exp Med 198: 623–634. 12925678 10.1084/jem.20030390 PMC2194172

[21] Klebanoff CA, Spencer SP, Torabi-Parizi P, (2013) Retinoic acid controls the homeostasis of pre-cDC-derived splenic and intestinal dendritic cells. J Exp Med 210: 1961–1976. 23999499 10.1084/jem.20122508 PMC3782040

[22] Beijer MR, Molenaar R, Goverse G, Mebius RE, Kraal G, (2013) A crucial role for retinoic acid in the development of Notch-dependent murine splenic CD8- CD4- and CD4+ dendritic cells. Eur J Immunol 43: 1608–1616. 23519987 10.1002/eji.201343325

[23] Saurer L, McCullough KC, Summerfield A (2007) In vitro induction of mucosa-type dendritic cells by all-trans retinoic acid. J Immunol 179: 3504–3514. 17785784 10.4049/jimmunol.179.6.3504

[24] Zeng R, Oderup C, Yuan R, Lee M, Habtezion A, (2013) Retinoic acid regulates the development of a gut-homing precursor for intestinal dendritic cells. Mucosal Immunol 6:847–856. 23235743 10.1038/mi.2012.123 PMC3612556

[25] Mohty M, Morbelli S, Isnardon D, Sainty D, Arnoulet C, (2003) All-trans retinoic acid skews monocyte differentiation into interleukin-12-secreting dendritic-like cells. Br J Haematol 122: 829–836. 12930397 10.1046/j.1365-2141.2003.04489.x

[26] Hengesbach LM, Hoag KA (2004) Physiological concentrations of retinoic acid favor myeloid dendritic cell development over granulocyte development in cultures of bone marrow cells from mice. J Nutr 134: 2653–2659. 15465762 10.1093/jn/134.10.2653

[27] Horowitz MM, Gale RP, Sondel PM, Goldman JM, Kersey J, (1990) Graft-versus-leukemia reactions after bone marrow transplantation. Blood 75: 555–562. 2297567

[28] Shlomchik WD (2007) Graft-versus-host disease. Nat Rev Immunol 7: 340–352. 17438575 10.1038/nri2000

[29] Welniak LA, Blazar BR, Murphy WJ (2007) Immunobiology of allogeneic hematopoietic stem cell transplantation. Annu. Rev. Immunol 25: 139–170. 17129175 10.1146/annurev.immunol.25.022106.141606

[30] Choi SW, Reddy P (2014) Current and emerging strategies for the prevention of graft-versus-host disease. Nat Rev Clin Oncol 11: 536–547. 24958183 10.1038/nrclinonc.2014.102 PMC4151470

[31] Hill GR, Ferrara JLM (2000) The primacy of the gastrointestinal tract as a target organ of acute graft-versus-host disease: rationale for the use of cytokine shields in allogeneic bone marrow transplantation. Blood 95: 2754–2759. 10779417

[32] Hill GR, Crawford JM, Cooke KR, Brinson YS, Pan L, (1997) Total body irradiation and acute graft versus host disease: the role of gastrointestinal damage and inflammatory cytokines. Blood 90: 3204–3213. 9376604

[33] Shlomchik WD, Couzens MS, Tang CB, McNiff J, Robert ME, (1999) Prevention of graft versus host disease by inactivation of host antigen-presenting cells. Sci 285: 412–415. 10.1126/science.285.5426.412 10411505

[34] Zhang Y, Shlomchik WD, Joe G, Louboutin JP, Zhu J, (2002) APCs in the liver and spleen recruit activated allogeneic CD8^+^ T cells to elicit hepatic graft-versus-host disease. J Immunol 169: 7111–7118. 12471148 10.4049/jimmunol.169.12.7111

[35] Koenecke C, Prinz I, Bubke A, Schreder A, Lee CW, (2012) Shift of graft versus host disease organ tropism by dietary vitamin A. Plos One 7: 1–9. 10.1371/journal.pone.0038252 PMC3364223 22666498

[36] Chen X, Dodge J, Komorowski R, Drobyski WR (2013) A critical role for the retinoic acid signaling pathway in the pathophysiology of gastrointestinal graft-versus-host disease. Blood 121: 3970–3980. 23529927 10.1182/blood-2012-08-445130 PMC3650708

[37] Aoyama K, Saha A, Tolar J, Riddle MJ, Veenstra RG, (2013) Inhibiting retinoic acid signaling ameliorates graft-versus-host disease by modifying T-cell differentiation and intestinal migration. Blood 122: 2125–2134. 23814022 10.1182/blood-2012-11-470252 PMC3778553

[38] Wang D, Yu Y, Haarberg K, Fu J, Kaosaard K, (2013) Dynamic change and impact of myeloid-derived suppressor cells in allogeneic bone marrow transplantation in mice. Biol Blood Marrow Transplant 19: 692–702. 23376089 10.1016/j.bbmt.2013.01.008 PMC4011929

[39] Dodge J, Stephans A, Lai J, Drobyski WR, Chen X (2016) Effects of donor vitamin A deficiency and pharmacologic modulation of donor t cell retinoic acid pathway on the severity of experimental graft-versus-host disease. Biol Blood Marrow Transplant 22: 2141–2148. 27596131 10.1016/j.bbmt.2016.09.001

[40] Lounder DT, Khandelwal P, Dandoy CE, Jodele S, Grimley MS, (2017) Lower levels of vitamin A are associated with increased gastrointestinal graft-versus-host disease in children. Blood 129: 2801–2807. 28279965 10.1182/blood-2017-02-765826 PMC5766846

[41] Tong J, Hu R, Zhao Y, Xu Y, Zhao X, (2017) Serum vitamin Alevels may affect the severity of ocular graft-versus-host disease. Front. Med 4: 67. 10.3389/fmed.2017.00067 PMC5479876 28691006

[42] Carpenter PA (2017) Vitamin A to reduce gut leak and GVHD? Blood 129: 2715–2717. 28522464 10.1182/blood-2017-03-773226

